# Awassi Ancestry-Associated Serum Metabolomic Signatures Reveal Immune–Metabolic Features Related to Health Robustness in Dairy Sheep

**DOI:** 10.3390/vetsci13080796

**Published:** 2026-08-11

**Authors:** Jianqing Zhao, Wei Wang, Yuzhu Guo, Shuyu Shi, Yuanpan Mu, Jiayidaer Kamalibieke, Chenbo Shi, Lei Shi, Jun Luo

**Affiliations:** 1 Shaanxi Key Laboratory of Molecular Biology for Agriculture, College of Animal Science and Technology, Northwest A&F University, Yangling 712100, China; 2 College of Animal Science, Xinjiang Agricultural University, Urumqi 830052, China; 3 Shanxi Fourteen Sheep Seed Industry Co., Ltd., Jinzhong 031800, China

**Keywords:** Awassi Dairy Sheep, crossbreeding, disease resilience, immune metabolism, lipid metabolism, serum metabolomics

## Abstract

Genetic background can shape metabolic phenotypes and may contribute to differences in health robustness among livestock breeds. This study investigated Awassi-associated serum metabolic characteristics in dairy sheep by integrating untargeted metabolomic profiling with farm-level morbidity information. Serum samples were collected from 36 adult sheep representing six genetic groups raised under common feeding and management conditions, Awassi, East Friesian, Hu, and three Awassi–East Friesian–Hu crossbred groups, with six animals per group and an equal number of males and females. Following data preprocessing and normalization, 2284 metabolic features were retained for comparative analysis. Principal component analysis revealed marked differences in serum metabolic profiles among the genetic groups, with Awassi sheep displaying a distinct metabolic pattern relative to the purebred and crossbred populations. Across five comparisons between Awassi and the other genetic groups, 17 metabolic features were consistently more abundant in Awassi sheep. These features were mainly associated with lipid mediator metabolism, glycerophospholipid metabolism, aromatic and indole-related metabolism, and host–microbial co-metabolism. A metabolic signature derived from the Awassi–East Friesian comparison differed significantly among the six groups and was positively associated with Awassi ancestry at the individual level. However, the two crossbred groups with 50% Awassi ancestry did not show a simple intermediate metabolic pattern, suggesting that crossbred metabolic phenotypes may involve non-additive genetic effects. Farm records showed lower total morbidity in Awassi and the three crossbred populations than in the East Friesian and Hu populations. Because the morbidity data were summarized at the population level and were not individually matched to the metabolomic samples, they were interpreted as supporting production context rather than direct metabolite–disease associations. Collectively, these results reveal a distinctive Awassi-associated serum metabolic signature and provide new insights into breed- and ancestry-related metabolic variation in dairy sheep.

## Simple Summary

Improving the health and adaptability of dairy sheep is important for sustainable production. Awassi sheep are well known for their ability to perform under challenging environmental conditions, but the biological basis of this robustness is not fully understood. In this study, we compared serum metabolic profiles among Awassi, East Friesian, Hu, and three crossbred sheep populations raised under the same feeding and management conditions. Awassi sheep showed a clearly different metabolic pattern, particularly in metabolites related to lipid metabolism, membrane components, and interactions between the animal and its microbial environment. The crossbred groups showed distinct metabolic characteristics and did not simply follow the proportion of Awassi ancestry, suggesting that crossbreeding may produce more complex biological effects. Farm health records also showed lower morbidity in Awassi and crossbred populations than in East Friesian and Hu sheep. These findings improve our understanding of breed-related metabolic differences and may support future research on the selection of healthier and more adaptable dairy sheep.

## 1. Introduction

Improving disease resilience while maintaining productivity is a major goal in modern livestock breeding. Disease resilience is conceptually distinct from disease resistance: resistance refers to the capacity to limit pathogen burden, whereas resilience includes the ability to maintain health and performance under infectious or environmental challenge [1,2,3]. Operational resilience phenotypes are increasingly discussed at both animal and herd levels, but robust inference ideally requires longitudinal health, performance, or challenge-response records [4]. In the absence of pathogen-load measurements or individually matched clinical records, metabolomics can identify baseline metabolic states potentially related to resilience biology, but it cannot independently establish disease resistance or resilience [1,3,4].

Dairy sheep production increasingly relies on breed selection and crossbreeding to combine milk yield, adaptation, fertility, and robustness. Awassi sheep are an important Middle Eastern fat-tailed dairy breed known for adaptation across diverse production systems and for their use in dairy-oriented genetic improvement programs [5]. East Friesian sheep are widely used for dairy performance, while Hu sheep contribute fecundity and adaptation traits in Chinese production systems [6]. Recent sheep microbiome–metabolome studies further suggest that breed background can shape intestinal or serum metabolic profiles under comparable feeding conditions [7,8]. The combination of pure breeds and crossbred groups therefore provides an opportunity to evaluate whether ancestry composition is associated with serum metabolic profiles under a common environment.

Serum metabolomics can capture systemic variation in lipid, amino acid, and purine metabolism, as well as host–microbial co-metabolism and inflammatory lipid signaling. Studies in sheep and other ruminants have shown that serum or plasma metabolomic profiles reflect nutritional status, feed efficiency, grazing pressure, developmental stage and host–microbial interactions [7,8,9,10]. Lipid mediators, phospholipid and sphingolipid metabolism, tryptophan/indole metabolism, and purinergic pathways contribute to immune cell activation, inflammatory resolution, oxidative stress responses, membrane organization, and host–microbiome communication [11,12,13,14,15,16,17,18,19]. Accordingly, comprehensive serum metabolomic profiling provides a useful approach for identifying breed-associated metabolic characteristics and for exploring biological pathways potentially related to adaptation and health robustness in dairy sheep.

The present study used a non-targeted UPLC-QTOF/MS serum metabolomics dataset from six adult dairy sheep genetic groups raised under common feeding and management conditions and integrated it with farm-level morbidity records. The specific objectives were to: (i) determine whether global serum metabolomic profiles differed among the six groups; (ii) identify metabolites consistently enriched in Awassi sheep relative to purebred and crossbred comparators; (iii) evaluate whether an Awassi-associated signature was reflected in crossbred groups; and (iv) interpret the results within a disease resilience-related immune–metabolic framework while clearly distinguishing population-level morbidity context from individual-level metabolite–phenotype associations.

## 2. Materials and Methods

### 2.1. Experimental Animals and Study Design

A total of 36 clinically healthy adult sheep were selected from six genetic groups, with six animals per group: Awassi (A), East Friesian (D), Hu (H), and three Awassi–East Friesian–Hu crossbred groups designated L, T, and S. Each group included three males and three females to maintain a balanced sex composition across the genetic groups. All animals were housed in the same sheep facility and were maintained under identical feeding, husbandry, and environmental conditions. Blood samples were collected in the morning before feeding.

The number of animals included in each group was determined according to the availability of eligible animals, the requirement for independent biological replication in untargeted serum metabolomics, and the need to maintain a balanced experimental design among the six genetic groups. Because only three animals of each sex were included within each group, sex-specific metabolic effects and sex-by-genetic-group interactions were not analyzed separately.

The ancestry composition of the six groups is presented in Table 1. The purebred groups comprised 100% Awassi, East Friesian, or Hu ancestry. The three crossbred groups differed in their breed composition: L contained 25.0% Awassi, 62.5% East Friesian, and 12.5% Hu ancestry; T contained 50.0% Awassi, 25.0% East Friesian, and 25.0% Hu ancestry; and S contained 50.0% Awassi, 37.5% East Friesian, and 12.5% Hu ancestry. The three crossbred populations were analyzed as separate genetic groups according to their distinct ancestry compositions.

### 2.2. Serum Collection and UPLC-QTOF/MS Metabolomics

Blood samples were collected from healthy adult sheep using vacuum blood collection tubes without anticoagulant (or serum separator tubes). After clotting at room temperature for 30 min, serum was separated by centrifugation at 3000× *g* for 15 min at 4 °C. The supernatant was aliquoted and stored at −80 °C until metabolomic analysis. For metabolite extraction, 100 µL of serum was mixed with 400 µL of ice-cold methanol containing internal standards (e.g., L-phenylalanine-d5 and lysophosphatidylcholine-19:0), vortexed, and centrifuged at 14,000× *g* for 10 min at 4 °C. The supernatant was transferred to a clean vial and dried under nitrogen; the residue was reconstituted in 100 µL of acetonitrile/water (1:1, *v*/*v*) and filtered through a 0.22 µm membrane prior to injection. Non-targeted metabolomic profiling was performed using ultra-performance liquid chromatography coupled with quadrupole time-of-flight mass spectrometry (UPLC-QTOF/MS; Waters Corporation, Milford, MA, USA). According to the available methods information, chromatographic separation was performed on a Waters Acquity I-Class PLUS UPLC system (Waters Corporation, Milford, MA, USA) equipped with a Waters ACQUITY UPLC HSS T3 column (2.1 × 100 mm, 1.8 µm; Waters Corporation, Milford, MA, USA) maintained at 40 °C. The mobile phase consisted of water with 0.1% formic acid (A) and acetonitrile with 0.1% formic acid (B). The gradient program was: 0–1 min, 5% B; 1–11 min, 5–95% B; 11–13 min, 95% B; 13–13.1 min, 95–5% B; followed by 2 min re-equilibration at 5% B. The flow rate was 0.35 mL/min, and the injection volume was 2 µL. Mass spectrometric detection was carried out on a Xevo G2-XS QTOF mass spectrometer (Waters Corporation, Milford, MA, USA) operating in both positive and negative electrospray ionization modes. The ion source parameters were: capillary voltage 2.5 kV (positive)/2.0 kV (negative), cone voltage 40 V, desolvation temperature 450 °C, source temperature 120 °C, desolvation gas flow 800 L/h, and cone gas flow 50 L/h. Data were acquired in full-scan mode over a mass range of 50–1200 *m*/*z* with a scan time of 0.2 s. Collision energy was ramped from 20 to 45 eV for MS/MS fragmentation. To ensure analytical stability, pooled quality control (QC) samples were prepared by mixing equal volumes of all individual serum extracts; one QC sample was injected after every 10 study samples, and the injection order of study samples was randomized.

### 2.3. Metabolomic Data Preprocessing and Analytical Quality Control

Raw LC–MS data acquired using MassLynx V4.2 were processed in Progenesis QI for peak detection, retention-time alignment, and feature extraction [20,21,22,23]. The original peak-area data were normalized using total peak-area normalization before subsequent statistical analysis. After data preprocessing and quality filtering, 2284 metabolic features from the 36 biological serum samples were retained for the comparative analyses. Metabolite annotation was performed in Progenesis QI v3.1 by integrating accurate-mass information, chromatographic retention characteristics, MSE-derived precursor- and fragment-ion information, and theoretical fragment recognition [24]. Candidate metabolite identities were matched against the online METLIN database, relevant public databases, and Biomark’s in-house library. The annotated metabolites were subsequently searched against the KEGG, HMDB, and LIPID MAPS databases to obtain chemical-class and pathway information. Pooled quality control samples were prepared by combining equal aliquots from the study samples and were analyzed together with the biological samples. Analytical stability and sample reproducibility were evaluated using principal component analysis and Spearman correlation analysis [25,26]. For the present analysis, metabolite abundance values were log2-transformed as log2(x + 1), and feature-wise standardization was applied before principal component analysis. Metabolite annotations were interpreted according to the available accurate-mass, chromatographic, and fragmentation evidence, with particular caution applied to unexpected compound annotations.

### 2.4. Differential Metabolite and Signature Analyses

Pairwise differential metabolite analyses were performed for all 15 comparisons among the six genetic groups. Fold change was calculated from normalized metabolite abundances, and between-group differences were assessed using Student’s *t*-test. Orthogonal partial least-squares discriminant analysis (OPLS-DA) was conducted using the R package ropls [27,28,29,30]. Model reliability was evaluated by 200 permutation tests, and VIP values were calculated through multiple cross-validation. Differential metabolites were screened using the combined criteria of FC > 1, *p* < 0.05, and VIP > 1. The reported *p* values were nominal values. For Awassi-centered comparisons, metabolites were classified as higher in Awassi or in the corresponding comparator group according to group mean abundance and log2 fold change direction. Metabolites consistently enriched in Awassi were defined as those identified in all five Awassi-centered comparisons and showing higher abundance in Awassi in each comparison. An Awassi-associated metabolic signature score was constructed from the differential metabolites identified between Awassi and East Friesian sheep. Metabolite abundances were standardized as z-scores across all biological samples, with positive directions assigned to metabolites higher in Awassi and negative directions assigned to those higher in East Friesian. The mean signed z-score was calculated for each animal. Group differences were evaluated using the Kruskal–Wallis test, followed by pairwise Mann–Whitney U tests. Associations between the signature score and Awassi ancestry were assessed using Spearman and Pearson correlation analyses.

### 2.5. Pathway Interpretation

KEGG annotation and enrichment outputs provided by the metabolomics service were used to support pathway-level interpretation. KEGG is a widely used biological knowledge base for functional interpretation of high-throughput molecular data [31,32]. Because detailed KEGG tables with metabolite identifiers, counts, and FDR values were not included in the current uploaded files, pathway results are interpreted conservatively. Pathways with few mapped metabolites or without FDR support were treated as hypothesis-generating rather than definitive pathway activation or suppression. This conservative approach is consistent with current recommendations for transparent metabolomics reporting and pathway enrichment interpretation [33,34,35].

### 2.6. Farm-Level Disease Phenotype Statistics

Farm-level morbidity data were obtained from routine production and health records maintained by the farm for the six genetic populations included in this study. The recorded disease categories included pneumonia, diarrhea, cough, skin disease, and parasitic disease, and the farm provided category-specific and total morbidity percentages for each population. These records represented population-level summaries and were not individually matched to the 36 sheep used for serum metabolomic analysis. Therefore, the morbidity data were used only as descriptive production-level information and were not included in individual metabolite–phenotype association analyses.

## 3. Results

### 3.1. Overall Structure of the Serum Metabolomics Dataset

**Figure 1:**
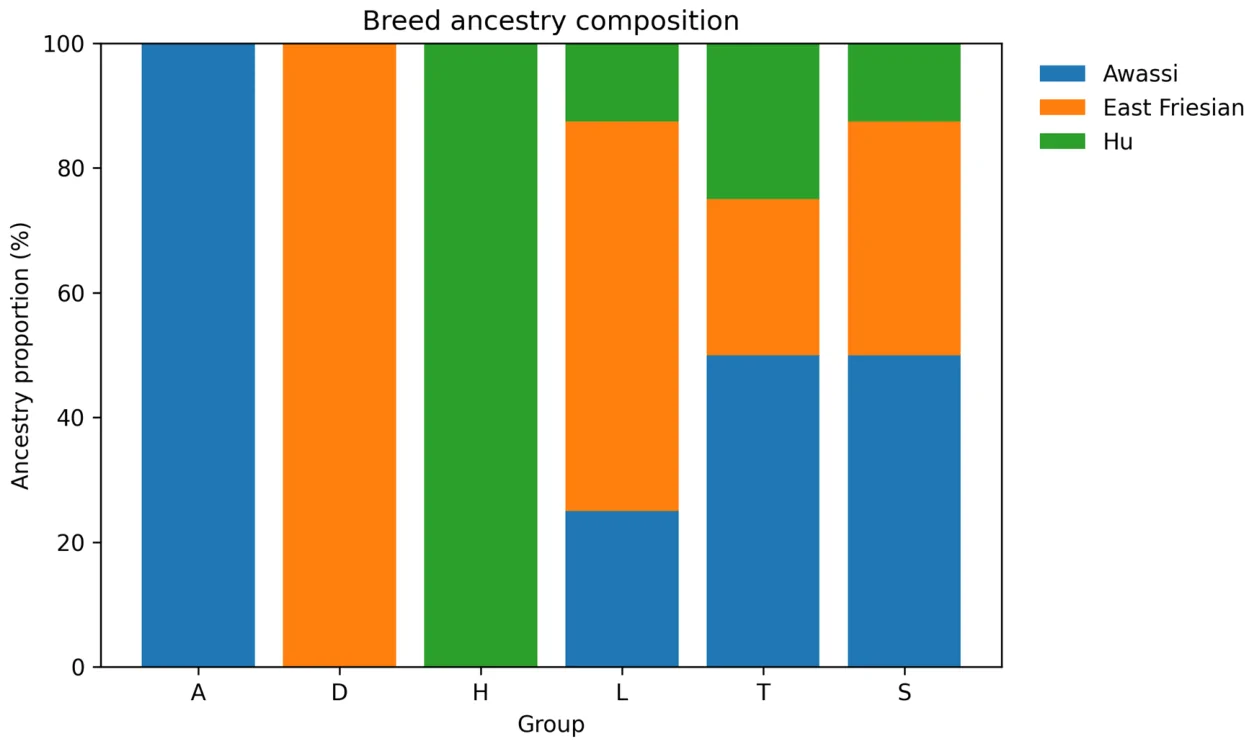
Ancestry composition of the six dairy sheep genetic groups used in this study.

**Figure 2:**
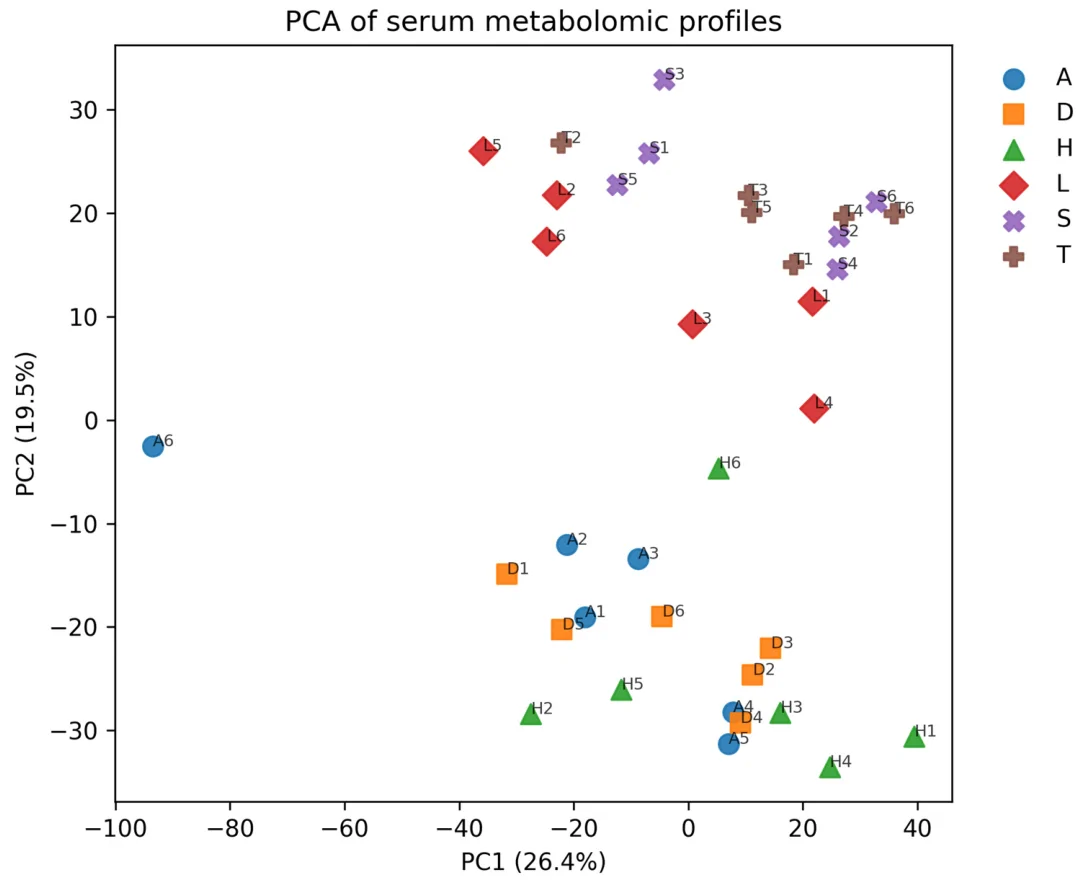
PCA of serum metabolomic profiles from 36 biological samples based on log2-transformed and feature-standardized normalized abundances. PC1 and PC2 explained 26.4% and 19.5% of the total variance, respectively.

The normalized abundance matrix comprised 2284 metabolite features from 36 biological serum samples and four pooled QC samples. The biological samples represented six genetic groups, namely Awassi (A), East Friesian (D), Hu (H), and three Awassi–East Friesian–Hu crossbred groups (L, T, and S), with six animals per group and a balanced sex structure of three males and three females in each group. PCA of log2-transformed and feature-standardized abundances showed that PC1 and PC2 explained 26.4% and 19.5% of the total variance, respectively, indicating breed- or ancestry-associated serum metabolic variation under common feeding and management conditions (Figure 1 and Figure 2).

### 3.2. Pairwise Differential Metabolite Profiles

**Figure 3:**
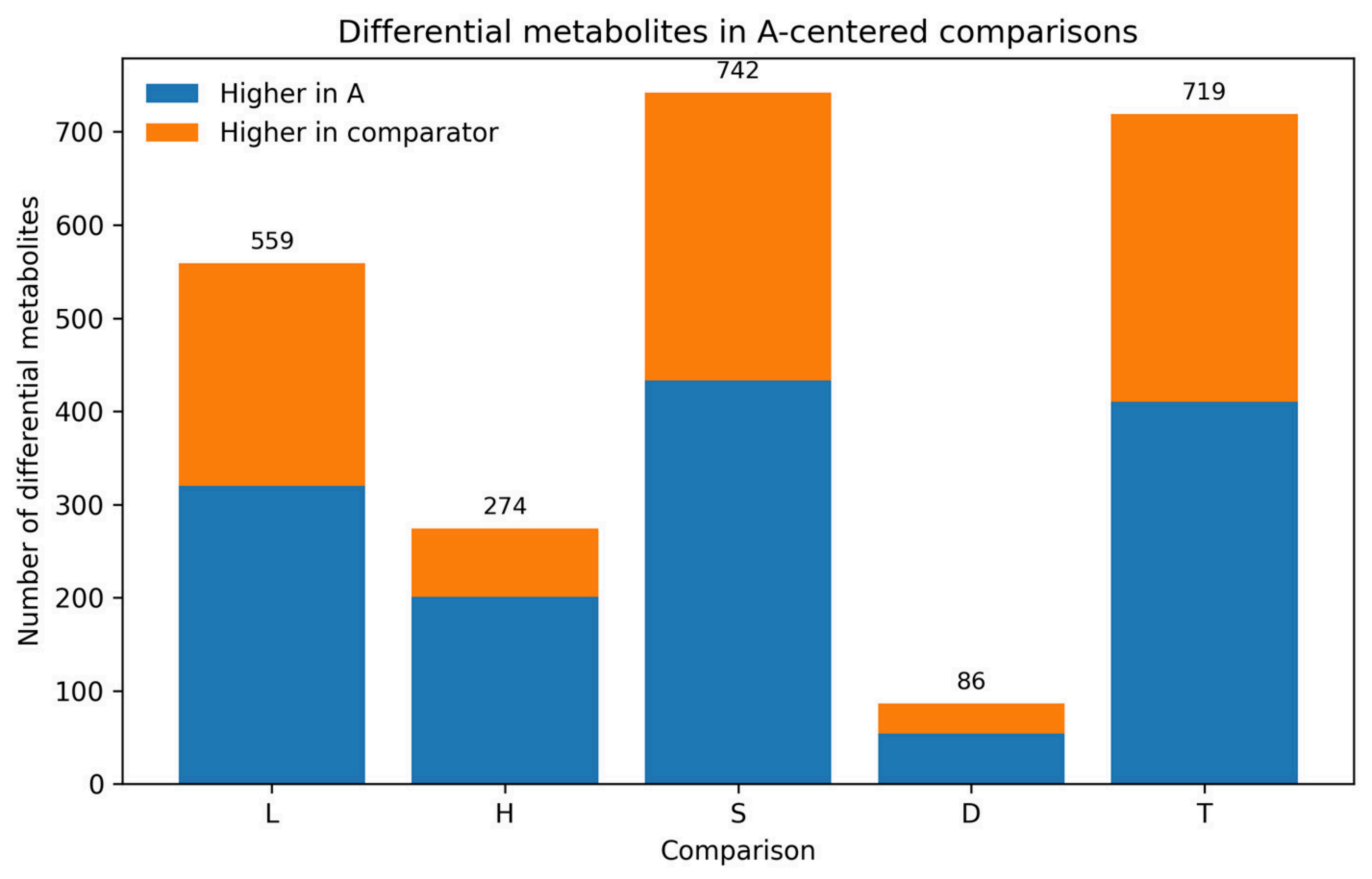
Differential metabolite counts in A-centered comparisons. Bars distinguish metabolites classified as higher in Awassi from metabolites classified as higher in the comparator group.

The number of differential metabolites varied substantially among pairwise comparisons (Table 2). Among A-centered comparisons, A_vs._D produced the smallest differential set, with 86 differential metabolites, including 54 higher in A and 32 higher in D. A_vs._H identified 274 differential metabolites, including 201 higher in A and 73 higher in H. Comparisons between A and the three crossbred groups yielded larger differential sets: 559 in A_vs._L (320 higher in A and 239 higher in L), 719 in A_vs._T (410 higher in A and 309 higher in T), and 742 in A_vs._S (433 higher in A and 309 higher in S). These results indicate that Awassi sheep were metabolically distinct not only from the two pure comparator breeds but also from the three crossbred groups (Figure 3).

### 3.3. Differential Metabolite Profile in the Awassi–East Friesian Comparison

**Figure 4:**
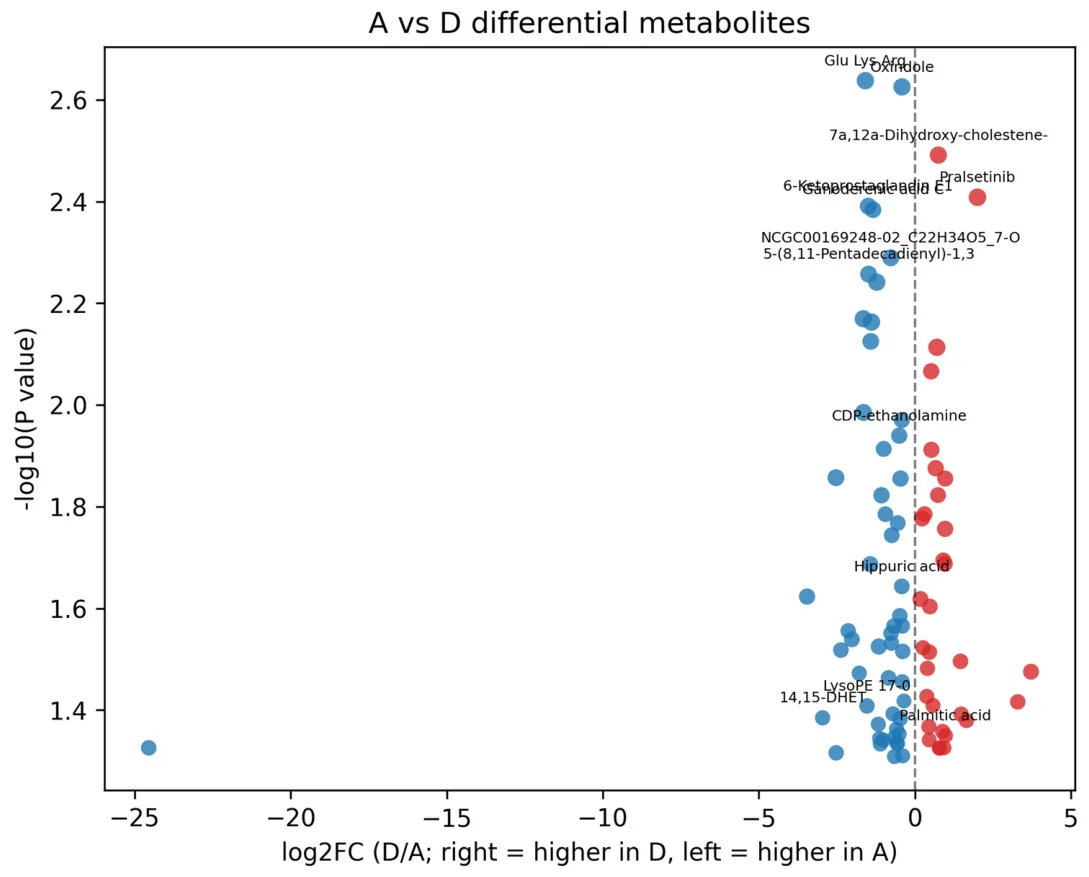
Volcano plot for the Awassi–East Friesian comparison. The x–axis shows log2 fold change (East Friesian/Awassi); therefore, negative values indicate higher abundance in Awassi and positive values indicate higher abundance in East Friesian. The y–axis shows −log10 of the nominal *p* value, and point size reflects the VIP score inherited from the service–generated supervised analysis.

The Awassi–East Friesian comparison identified 86 differential metabolites, including 54 metabolites higher in Awassi and 32 higher in East Friesian. Features higher in Awassi were putatively annotated as 14,15-DHET, inosine 5′-tetraphosphate, surfactin B, LysoPE 17:0, CDP-ethanolamine, hippuric acid and oxindole, whereas features higher in East Friesian were putatively annotated as N-palmitoyl proline, mauritine A, palmitic acid, trihexosylceramide (d18:1/18:1), palmitoyl glucuronide, and several putative xenobiotic or microbial secondary metabolite annotations. These annotation-level results suggest possible differences in lipid mediator-related, phospholipid-related, purine-related, and host–microbial co-metabolic features between Awassi and East Friesian sheep (Figure 4).

### 3.4. Core Awassi-Enriched Metabolites Across A-Centered Comparisons

**Figure 5:**
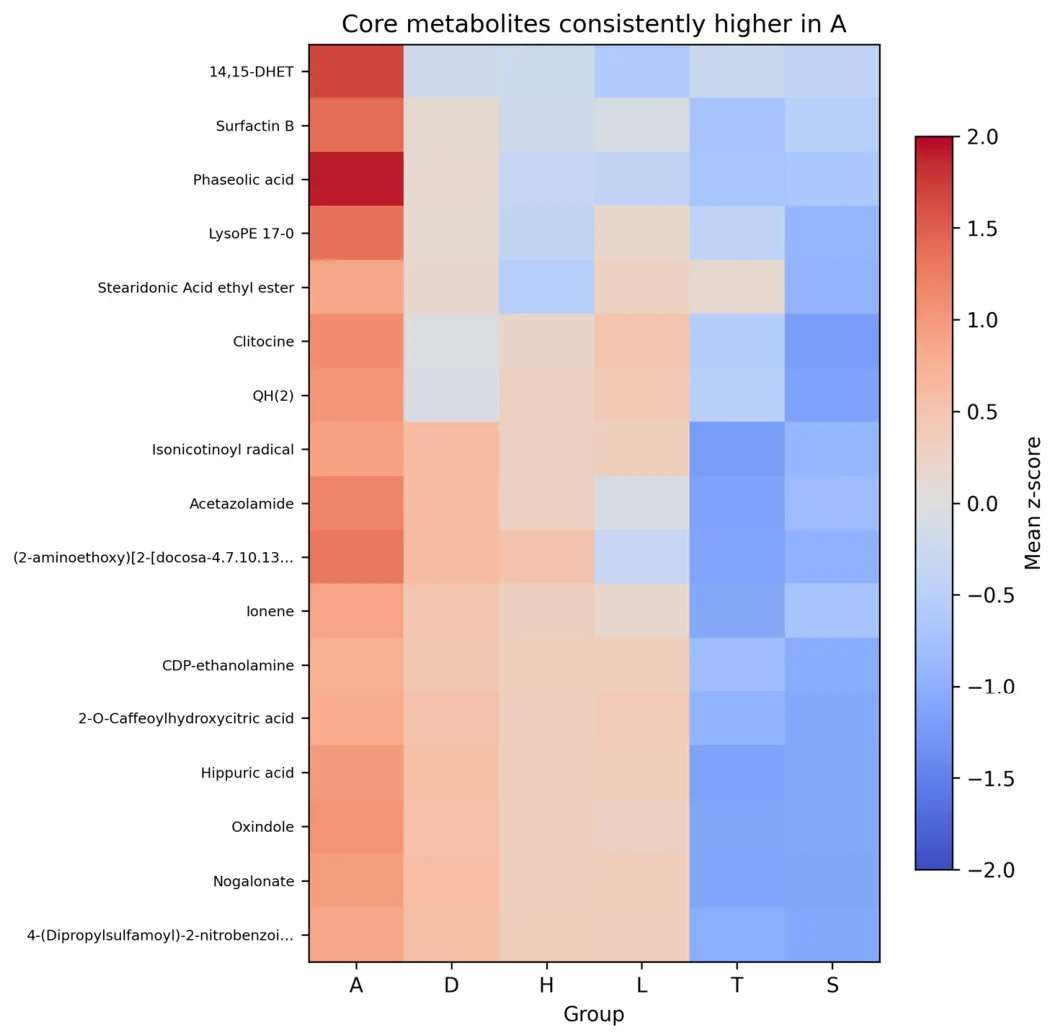
Heatmap of 17 core metabolites consistently higher in A across all A-centered comparisons. Values are group mean z-scores based on log2-transformed normalized abundance.

Intersection analysis across A_vs._D, A_vs._H, A_vs._L, A_vs._T, and A_vs._S identified 17 metabolites that were present in all five A-centered differential metabolite tables and consistently higher in A (Table 3). No metabolite was consistently lower in A across all five comparisons. The consistent Awassi-enriched features were putatively annotated as 14,15-DHET, stearidonic acid ethyl ester, LysoPE 17:0, CDP-ethanolamine, hippuric acid, oxindole, QH(2), phaseolic acid, clitocine, ionene, and several aromatic or lipid-like annotations. Collectively, these features defined an exploratory Awassi-enriched serum signature that separated A from D, H, and the three crossbred groups at the group mean level (Figure 5).

### 3.5. Awassi-Associated Signature Score and Crossbred Patterns

**Figure 6:**
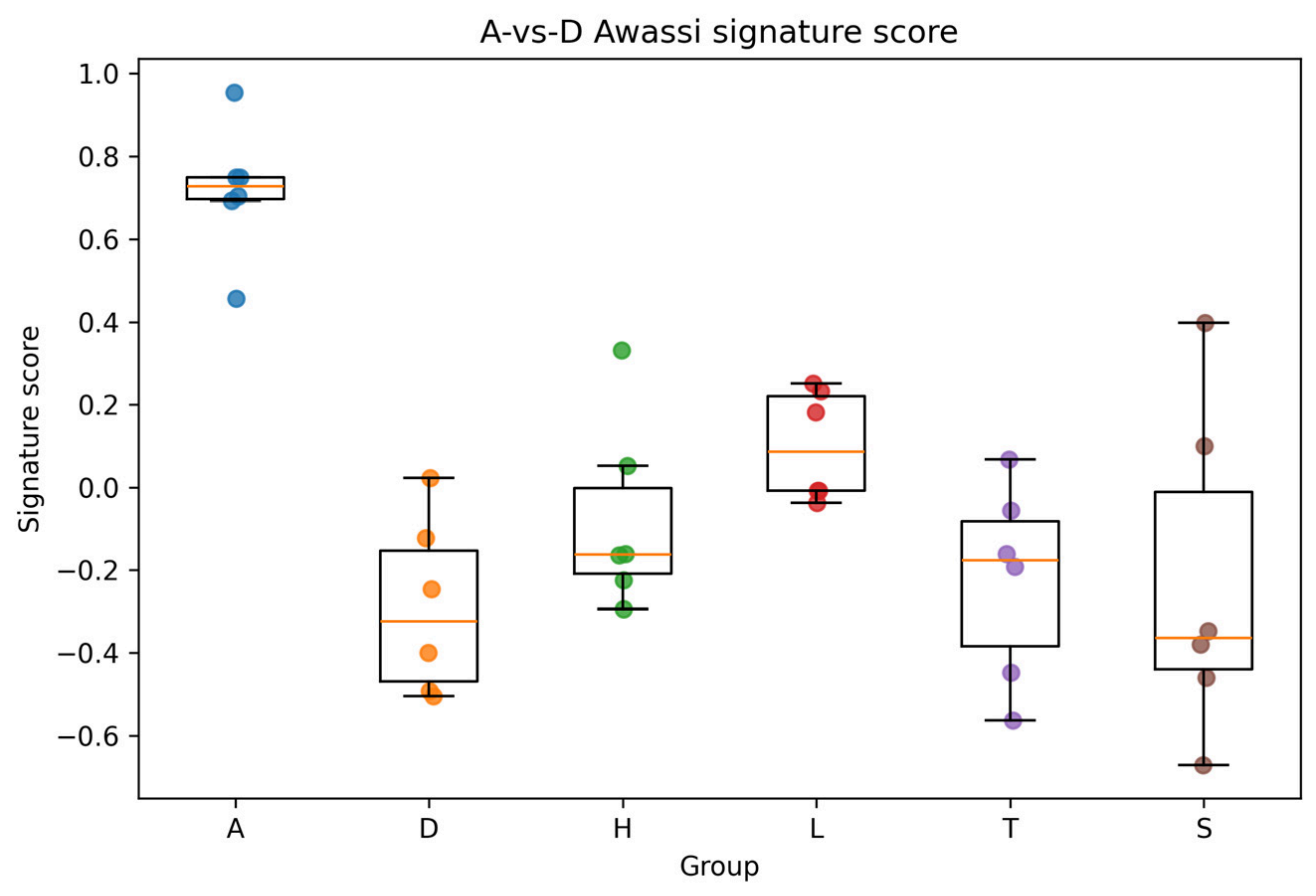
Awassi–East Friesian-derived metabolic signature score across the six groups. Positive values indicate a profile closer to A-enriched metabolites; negative values indicate a profile closer to D-enriched metabolites.

**Figure 7:**
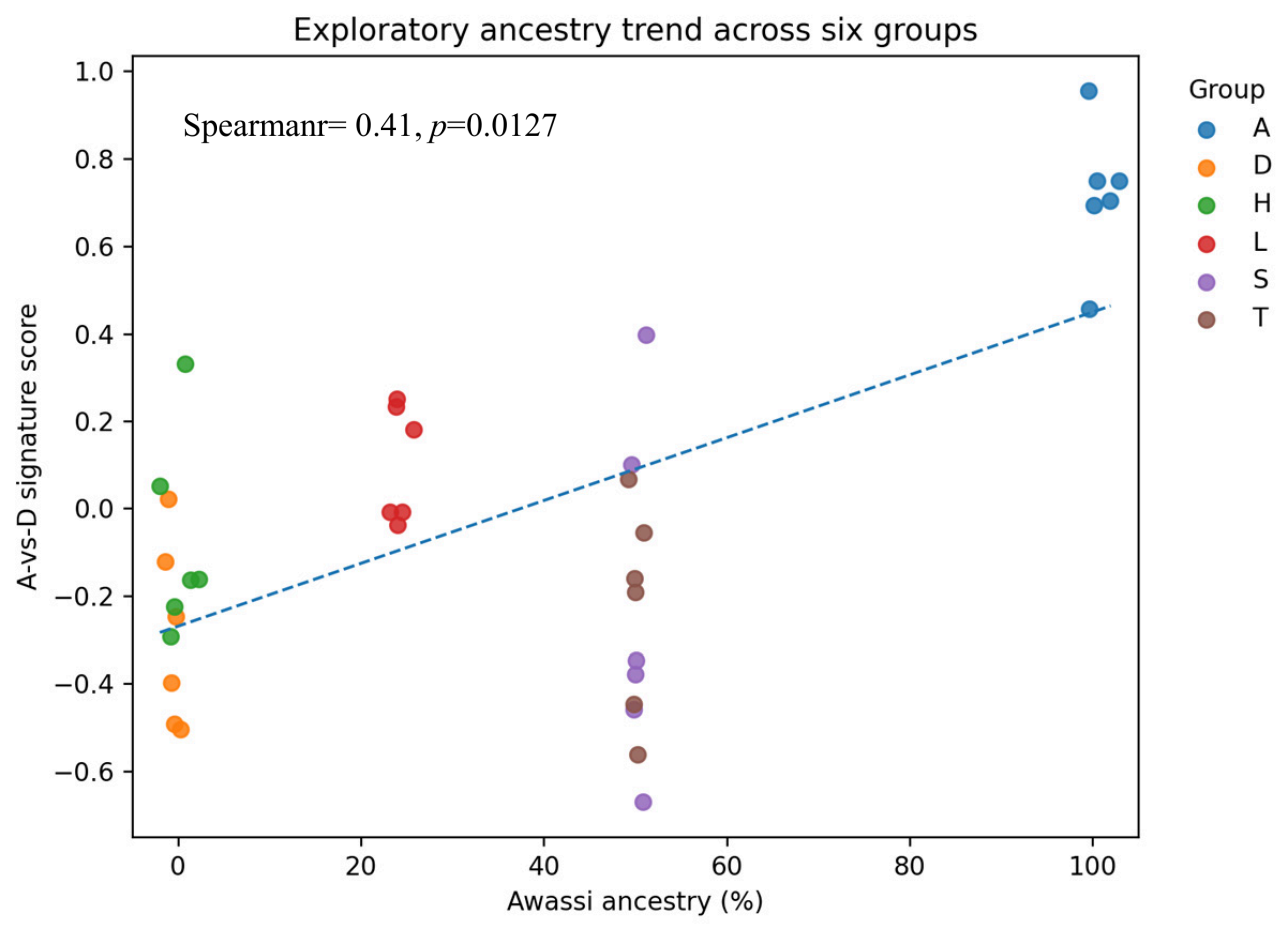
Exploratory relationship between Awassi ancestry percentage and A_vs_D-derived signature score across all six groups. The trend should be interpreted cautiously because ancestry levels are group-level variables and morbidity statistics were not individually matched to metabolomics samples.

To summarize Awassi–East Friesian-associated variation across all groups, an Awassi signature score was calculated from the 86 metabolites differing between Awassi and East Friesian after assigning positive weights to A-enriched metabolites and negative weights to D-enriched metabolites. This score differed among the six groups (Kruskal–Wallis *p* = 0.0012). The group mean score was highest in A (0.717), followed by L (0.102), H (−0.077), T (−0.225), S (−0.226), and D (−0.290) (Figure 6). Pairwise exploratory Mann–Whitney tests showed that A differed from each other group (all *p* = 0.0022). Across all 36 animals, the individual-level signature score was positively associated with Awassi ancestry (Spearman r = 0.411, *p* = 0.0127; Pearson r = 0.606, *p* = 9.06 × 10^−5^). However, group means showed that the two 50% Awassi crossbred groups did not simply move toward the A profile. This non-linear pattern suggests that crossbred serum metabolism may reflect non-additive ancestry interactions rather than a simple Awassi-dose response (Figure 7).

### 3.6. KEGG Annotation of Metabolites Differing Between Awassi and East Friesian

**Figure 8:**
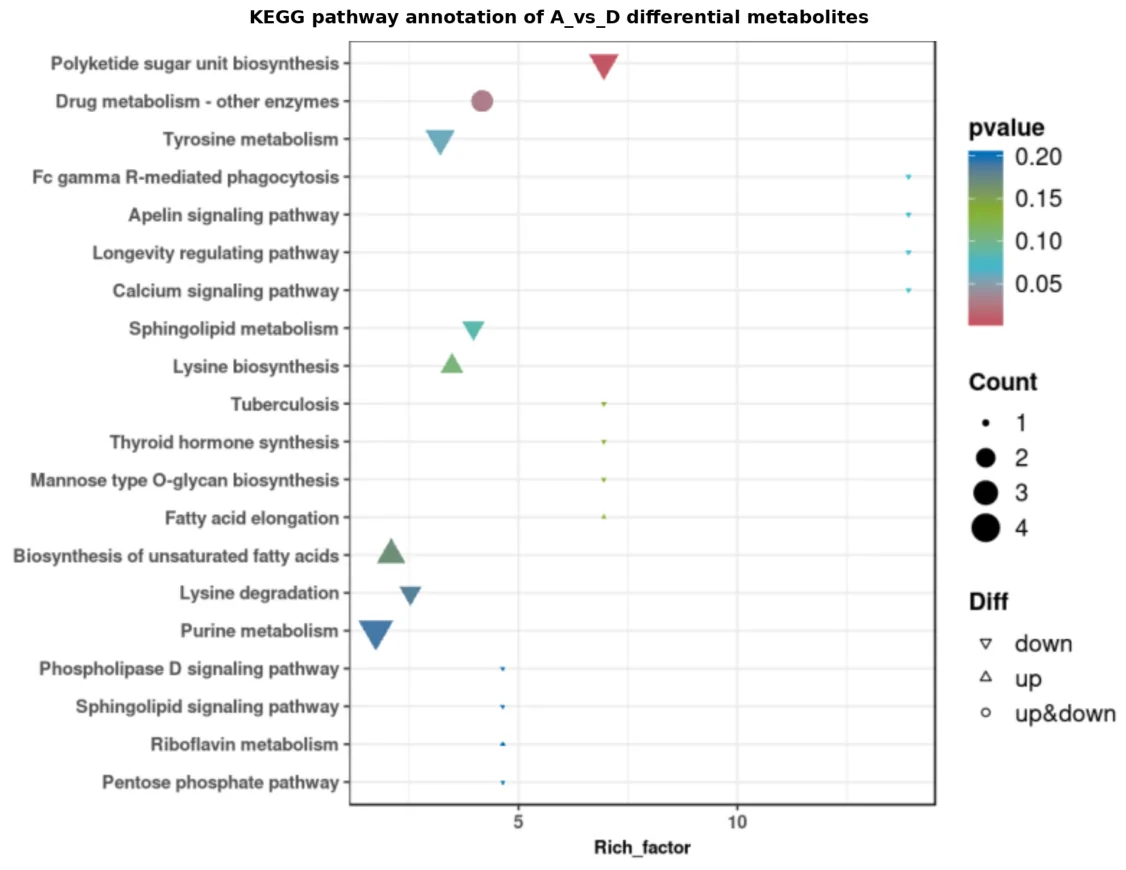
KEGG pathway annotation summary of metabolites differing between Awassi and East Friesian. The plot summarizes KEGG pathway categories associated with metabolites differing between Awassi and East Friesian. The x-axis indicates the Rich factor provided in the service-generated KEGG output, bubble size indicates the number of mapped differential metabolites, color indicates nominal *p* value, and shape indicates the direction category of the mapped metabolites.

KEGG annotation of the metabolites differing between Awassi and East Friesian mapped 11 metabolites to 12 pathways against a background of 320 annotated metabolites. The mapped pathways were mainly related to fatty acid metabolism, glycerophospholipid metabolism, biosynthesis of unsaturated fatty acids, arachidonic acid metabolism, purine metabolism, and riboflavin metabolism (Figure 8). Palmitic acid contributed to fatty acid-related annotations, CDP-ethanolamine mapped to glycerophospholipid metabolism, 14,15-DHET mapped to arachidonic acid metabolism, and inosine 5′-tetraphosphate mapped to purine metabolism. These mapped metabolites were consistent with the lipid, membrane phospholipid, eicosanoid-related, and purine-related signals observed at the metabolite level.

### 3.7. Farm-Level Morbidity Statistics

**Figure 9:**
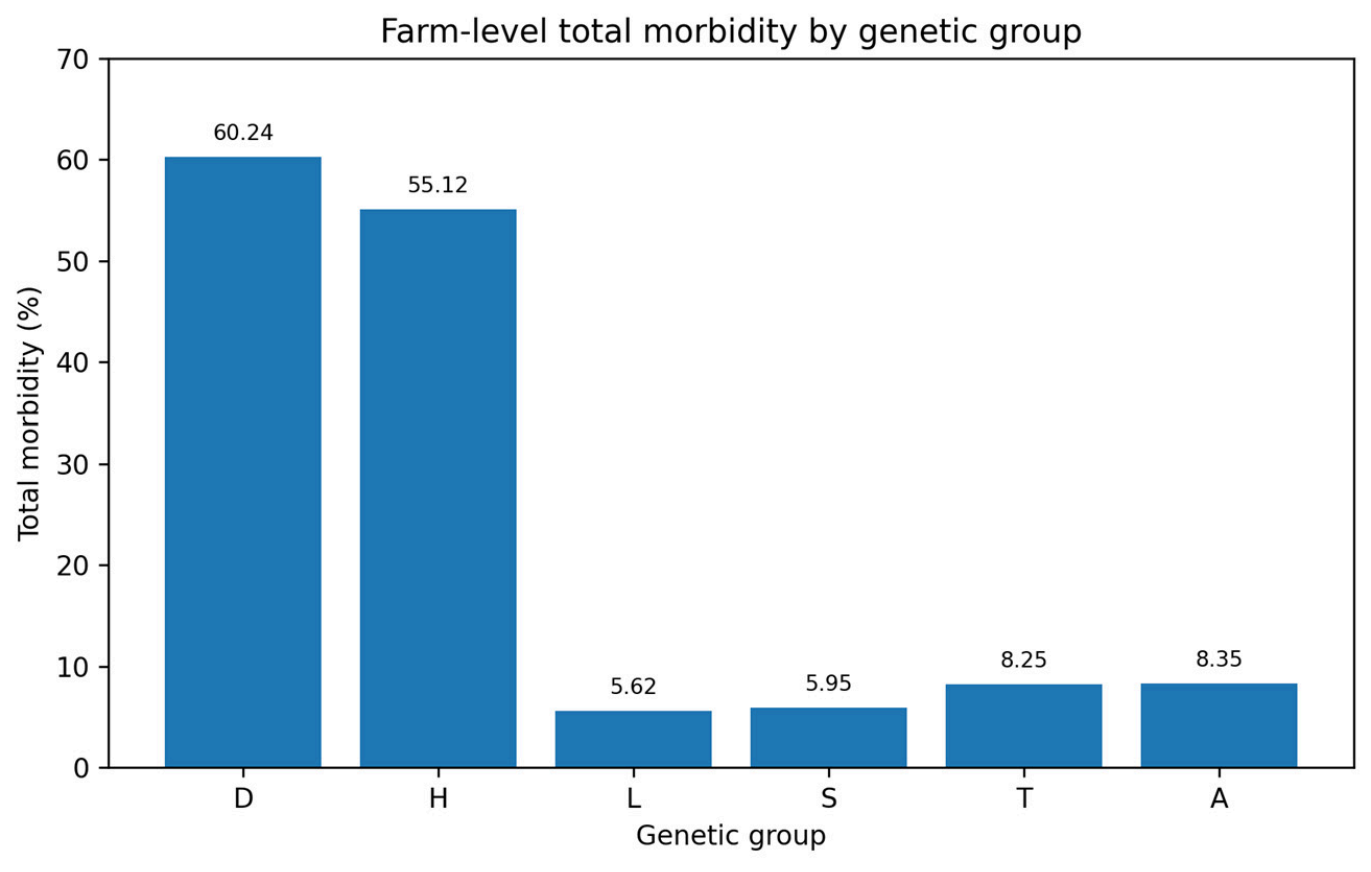
Farm-level total morbidity by genetic group. Morbidity values were summarized from production records and should be interpreted as population-level contextual phenotypes rather than individual-level disease outcomes matched to metabolomics samples.

Farm-level records showed higher total morbidity in East Friesian (D, 60.24%) and Hu (H, 55.12%) populations than in Awassi (A, 8.35%) and the three crossbred populations (L, 5.62%; T, 8.25%; S, 5.95%). Pneumonia, cough, and diarrhea were the main disease categories in D, whereas diarrhea, pneumonia, parasitic disease, and skin disease were prominent in H. In A and the crossbred populations, all individual disease category rates were below 2.5%, except parasitic disease in T (2.47%). Because these morbidity data were summarized at the population level and were not individually matched to the metabolomics animals, they were used only as descriptive production-level context rather than endpoints for metabolite–disease association testing (Figure 9).

## 4. Discussion

This study characterized serum metabolomic variation among six dairy sheep genetic groups raised under common feeding and management conditions, with a focus on Awassi-associated immune–metabolic features. Awassi sheep showed a distinct serum metabolic profile relative to East Friesian, Hu, and the three crossbred groups. In the A_vs._D comparison, 54 metabolites were higher in Awassi and 32 were higher in East Friesian, while intersection analysis identified 17 metabolites consistently higher in Awassi across all A-centered comparisons.

### 4.1. Awassi-Associated Lipid and Membrane-Metabolic Features

The 17-metabolite core signature suggests that Awassi sheep possess a distinctive baseline serum metabolic state under common management. Several core metabolites or annotations are related to lipid mediators and membrane lipid metabolism, including 14,15-DHET, stearidonic acid ethyl ester, LysoPE 17:0, and CDP-ethanolamine. Long-chain unsaturated fatty acids and their derivatives can modulate inflammation by changing membrane composition, cellular signaling, and lipid mediator production [11,36,37]. Specialized pro-resolving lipid mediators and eicosanoid-related metabolites also contribute to inflammatory resolution and host defense [12,38,39]. Phospholipid remodeling in innate immune cells can influence membrane architecture, antigen recognition, and inflammatory signaling [14]. Thus, higher abundance of selected lipid-related metabolites in A may indicate a distinct basal lipid-signaling environment rather than simply higher or lower inflammation.

### 4.2. Putative Host–Microbial Co-Metabolic Features

A second feature of the core signature involved aromatic, indole-like, or host–microbial co-metabolites, including hippuric acid and oxindole. In ruminants, serum metabolites are shaped not only by host genetics but also by rumen microbial metabolism, feed-derived compounds, absorption, and hepatic transformation. Microbial tryptophan catabolites and indole derivatives can regulate epithelial barrier function, aryl hydrocarbon receptor signaling, and immune homeostasis [15,16,40,41]. More broadly, gut microbiota-derived metabolites such as short-chain fatty acids, tryptophan derivatives, and bile acid metabolites influence host immunity and systemic homeostasis [42,43,44]. Under identical feeding and management, differences in host–microbial co-metabolites may therefore reflect breed-associated differences in rumen microbial ecology, gut–liver metabolism, or clearance. This interpretation is consistent with the appearance of KEGG categories related to secondary metabolites, xenobiotic/drug metabolism, and ABC transport, but it requires targeted validation and microbial data.

### 4.3. Non-Additive Patterns in Crossbred Groups

The crossbred groups added important information. Although the individual-level A_vs._D signature score correlated positively with Awassi ancestry across all 36 animals, the group means did not display a strict linear ancestry-dosage pattern. In particular, the two 50% Awassi groups did not show a simple intermediate or A-like pattern for the A_vs._D signature score. This observation suggests that the serum metabolome of crossbred sheep may be shaped by non-additive ancestry interactions, epistasis, breed complementarity, rumen–host metabolic interactions, or management-independent physiological differences, rather than by a single linear Awassi-proportion effect. Such non-linear responses are plausible in crossbreeding systems, where performance and robustness may be influenced by both additive ancestry and heterosis-related effects [5,6,7].

### 4.4. Interpretation of Population-Level Morbidity Context

Farm-level morbidity records provided an independent production context for the metabolomic findings. East Friesian and Hu populations showed high total morbidity (60.24% and 55.12%, respectively), whereas Awassi and the three crossbred populations showed markedly lower total morbidity (5.62–8.35%). This pattern is compatible with greater health robustness in Awassi-containing populations under the local farm environment. However, resilience is a production-level and challenge-dependent phenotype, and these records cannot establish pathogen resistance or causal metabolite–disease relationships without pathogen burden, standardized clinical definitions, longitudinal individual records, and matched metabolomic phenotypes [1,2,4]. Therefore, the morbidity data were used to contextualize the metabolic results and to motivate future validation, rather than as direct evidence that any individual metabolite mediates disease protection.

**Figure 10:**
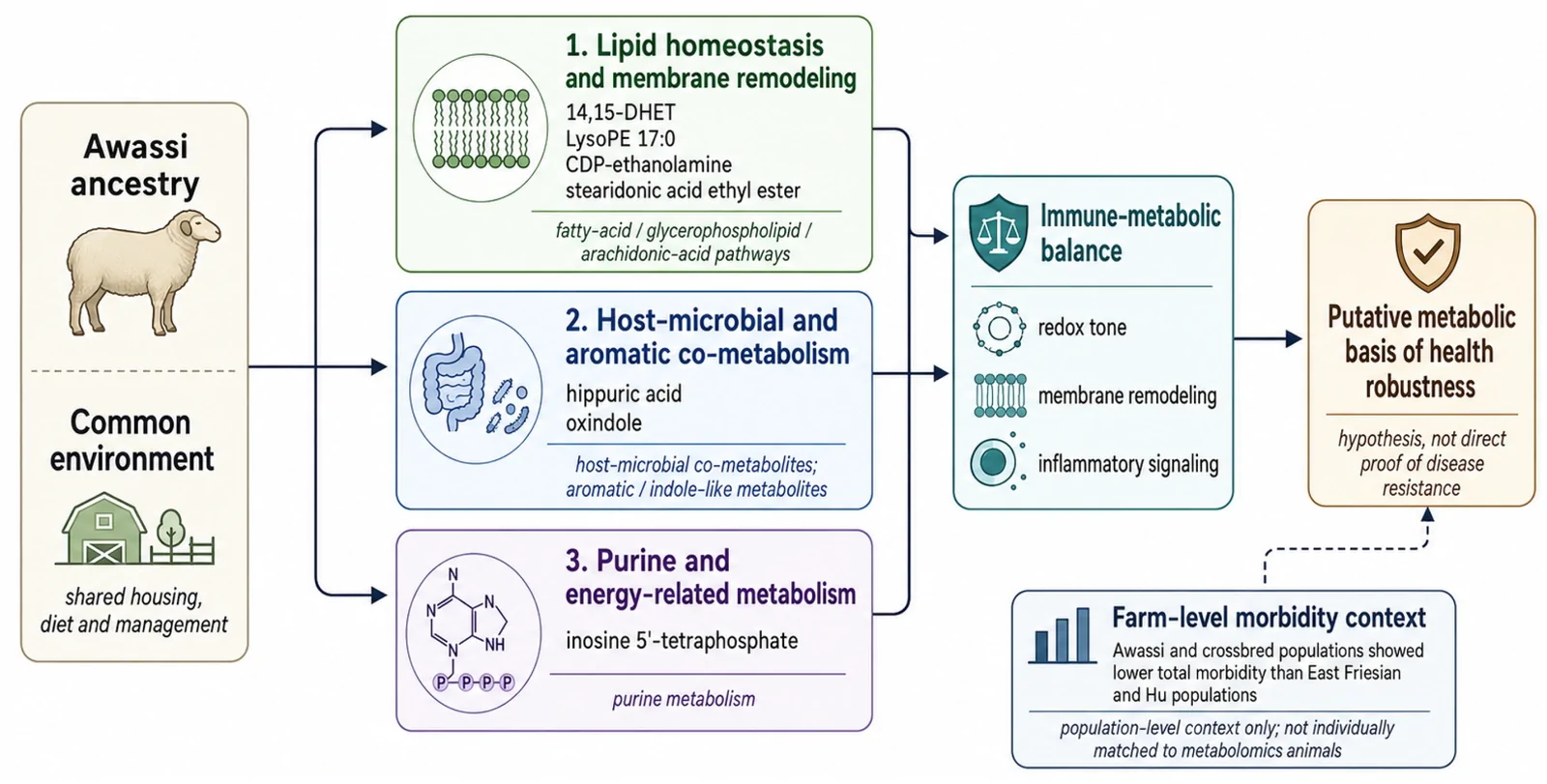
Conceptual model of the Awassi-associated immune–metabolic phenotype in dairy sheep.

Based on these findings, we propose a conceptual model in which Awassi ancestry is associated with coordinated differences in lipid homeostasis, host–microbial/aromatic co-metabolism, and purine-related metabolism, which may collectively contribute to immune–metabolic balance under common farm conditions (Figure 10). This model is hypothesis-generating and should be tested using targeted metabolomics, immune phenotyping, and individually matched health records.

The model summarizes the main interpretations derived from the untargeted serum metabolomics analysis and the farm-level morbidity context. Awassi ancestry, under a common environment, was associated with differences in lipid homeostasis and membrane remodeling, host–microbial and aromatic co-metabolism, and purine-related metabolism. These metabolic features may collectively contribute to immune–metabolic balance, including redox tone, membrane remodeling, and inflammatory signaling. Farm-level morbidity data provide contextual production support, but because they were not individually matched to the metabolomics animals, the proposed relationship with health robustness should be regarded as hypothesis-generating rather than direct evidence of disease resistance.

The study should be interpreted in light of several limitations. First, each metabolomics group contained six animals, which limited statistical power and increased sensitivity to individual variation. Second, although all groups were balanced for sex, the small number of males and females within each group precluded formal sex-stratified inference. Third, the available serum material did not allow for targeted validation of immune biomarkers, oxidative stress markers, or candidate metabolites. Fourth, the morbidity records were summarized at the population level and require future confirmation using standardized case definitions, defined observation periods, denominators, and individual linkage to metabolomic samples. Fifth, some untargeted annotations resembled pharmaceuticals, antibiotics, or plant/microbial secondary metabolites; these features should be regarded as putative unless confirmed by MS/MS quality, retention-time matching, and authentic standards.

These limitations are balanced by several design features that support cautious interpretation. All genetic groups were maintained under the same housing, feeding, and management conditions, reducing major environmental confounding. The inclusion of three pure breeds and three crossbred groups enabled comparison of pure breed differences with crossbred metabolic remodeling. The core metabolite set, signature score analysis, KEGG annotation, and farm-level morbidity context together support a testable hypothesis: Awassi ancestry is associated with a distinct baseline serum metabolic state involving lipid mediator-related, membrane phospholipid, host–microbial/aromatic, and purine-related features that may contribute to immune–metabolic stability and health robustness. This hypothesis should be validated in larger cohorts with targeted metabolomics and matched individual health phenotypes.

## 5. Conclusions

Under common feeding and management conditions, Awassi dairy sheep showed a distinctive serum metabolomic profile relative to East Friesian, Hu, and three Awassi–East Friesian–Hu crossbred groups. Seventeen metabolites were consistently higher in Awassi across all A-centered comparisons, and the A_vs._D-derived signature score captured Awassi-associated metabolic variation across individuals and genetic groups. These features point to differences in lipid mediator-related, membrane phospholipid, aromatic/indole-like, host–microbial co-metabolic, and purine-related serum metabolites. Farm-level morbidity records showed substantially lower total morbidity in Awassi and the crossbred populations than in East Friesian and Hu populations, providing production-level context for the health robustness hypothesis. Nevertheless, because morbidity data were not individually matched to metabolomics samples and targeted validation assays were not available, the findings should be interpreted as hypothesis-generating evidence for a potential immune–metabolic basis of Awassi-associated health robustness rather than direct proof of enhanced disease resistance. Future work should integrate individual disease records, immune biomarkers, targeted metabolomics, and larger populations to validate these candidate signatures.

## Figures and Tables

**Table 1:** Experimental groups and ancestry composition.

Group	Genetic Group	*n*	Awassi %	East Friesian %	Hu %	Sex Structure
A	Awassi	6	100	0	0	3M/3F
D	East Friesian	6	0	100.000	0	3M/3F
H	Hu	6	0	0	100.000	3M/3F
L	East-Awassi-Hu	6	25	62.500	12.500	3M/3F
T	Awassi-East-Hu 2	6	50	25.000	25.000	3M/3F
S	Awassi-East-Hu 3	6	50	37.500	12.500	3M/3F

**Table 2:** Differential metabolite counts in A-centered comparisons. Metabolites were summarized as higher in Awassi or higher in the comparator group according to group mean abundance.

Comparison	Total_DEM	Higher in A	Higher in Comparator	Median log2FC	Minimum *p* Value	Median VIP
A_vs_L	559	320	239	−0.377	2.42 × 10^−11^	1.661
A_vs_H	274	201	73	−0.831	1.66 × 10^−5^	1.556
A_vs_S	742	433	309	−0.462	8.19 × 10^−9^	1.517
A_vs_D	86	54	32	−0.490	0.002	2.102
A_vs_T	719	410	309	−0.391	8.02 × 10^−10^	1.472

**Table 3:** Core metabolites consistently higher in A across all A-centered comparisons.

ID	Name	A_vs._D	A_vs._H	A_vs._L	A_vs._T	A_vs._S	A_vs._D_Pvalue	A_vs._D_VIP
pos_9725	14,15-DHET	−2.955	−3.076	−27.327	−3.546	−4.934	0.041	2.007
pos_10715	Surfactin B	−2.367	−3.680	−3.404	−5.203	−4.772	0.030	2.098
pos_5094	Phaseolic acid	−1.643	−2.962	−2.978	−3.369	−3.361	0.010	2.461
pos_9815	LysoPE 17-0	−1.533	−1.950	−1.563	−2.406	−2.938	0.039	2.062
pos_4619	Stearidonic Acid ethyl ester	−1.387	−2.025	−0.869	−1.079	−1.995	0.007	2.557
pos_5084	Clitocine	−0.996	−0.810	−0.654	−1.610	−2.071	0.012	2.178
pos_5088	QH(2)	−0.941	−0.766	−0.653	−1.516	−1.929	0.016	2.125
pos_1429	Isonicotinoyl radical	−0.699	−0.979	−0.872	−5.494	−3.179	0.040	1.955
pos_1132	Acetazolamide	−0.666	−1.106	−1.509	−2.776	−2.035	0.027	2.107
pos_8059	(2-aminoethoxy)[2-[docosa-4.7.10.13.16.19-…	−0.648	−0.682	−1.499	−2.236	−1.983	0.049	1.981
pos_1501	Ionene	−0.575	−0.859	−1.033	−1.998	−1.931	0.046	1.906
pos_1460	CDP-ethanolamine	−0.500	−0.719	−0.695	−2.310	−1.902	0.011	2.280
pos_1440	2-O-Caffeoylhydroxycitric acid	−0.481	−0.852	−0.750	−2.874	−2.272	0.026	2.082
pos_1431	Hippuric acid	−0.417	−0.661	−0.621	−2.025	−1.776	0.023	2.116
pos_1452	Oxindole	−0.410	−0.583	−0.618	−1.657	−1.576	0.002	2.559
pos_1441	Nogalonate	−0.402	−0.720	−0.677	−2.255	−1.972	0.027	2.069
pos_1439	4-(Dipropylsulfamoyl)-2-nitrobenzoic acid	−0.387	−0.686	−0.713	−2.372	−2.005	0.049	1.911

## Data Availability

The original contributions presented in this study are included in the article/Supplementary Materials. Further inquiries can be directed to the corresponding author(s).

## Supplementary Materials

The following supporting information can be downloaded at: https://www.mdpi.com/article/10.3390/vetsci13080796/s1. Supplementary Figure S1. PCA including QC samples. Supplementary Figure S2. Differential-metabolite counts across all 15 pairwise comparisons. Supplementary Figure S3. Core 17-metabolite Awassi-enriched signature score. Supplementary Figures S4. Volcano plot for A_vs_H after fold-change direction correction. Supplementary Figures S5. Volcano plot for A_vs_L after fold-change direction correction. Supplementary Figures S6. Volcano plot for A_vs_T after fold-change direction correction. Supplementary Figures S7. Volcano plot for A_vs_S after fold-change direction correction. Supplementary Figure S8. Disease-specific farm-level morbidity across genetic groups. Supplementary Figure S9. Group-level relationship between Awassi ancestry percentage and total morbidity. Supplementary Table S1. Normalized metabolite abundance matrix. Supplementary Table S2. Full differential-metabolite tables for all 15 comparisons. Supplementary Table S3. KEGG pathway annotation table with mapped metabolites for all 15 comparisons.
