# Supplementary material for: Awassi Ancestry-Associated Serum Metabolomic Signatures Reveal Immune–Metabolic Features Related to Health Robustness in Dairy Sheep
Source: Vet Sci. 2026 Aug 11;13(8):796. doi: 10.3390/vetsci13080796 (PMC13517851; doi:10.3390/vetsci13080796)
Supplement: Supplementary file 1 [file vetsci-13-00796-s001.zip › Supplementary_Figures.pdf]

Supplementary Materials

Awassi ancestry-associated serum metabolomic signatures reveal immune-metabolic features related to health robustness in dairy sheep

Jianqing Zhao[1,2], Wei Wang[2], Jiayidaer Kamalibieke[2], Shuyu Shi[3], Yuanpan Mu[2], Chenbo Shi[2], Lei Shi[3], Jun Luo[2*]

This supplementary file contains extended figure legends, supplementary figures and a list of accompanying supplementary tables. Spreadsheet tables are supplied as separate files in the Supplementary_Tables folder.

Supplementary Figures

Supplementary Figure S1

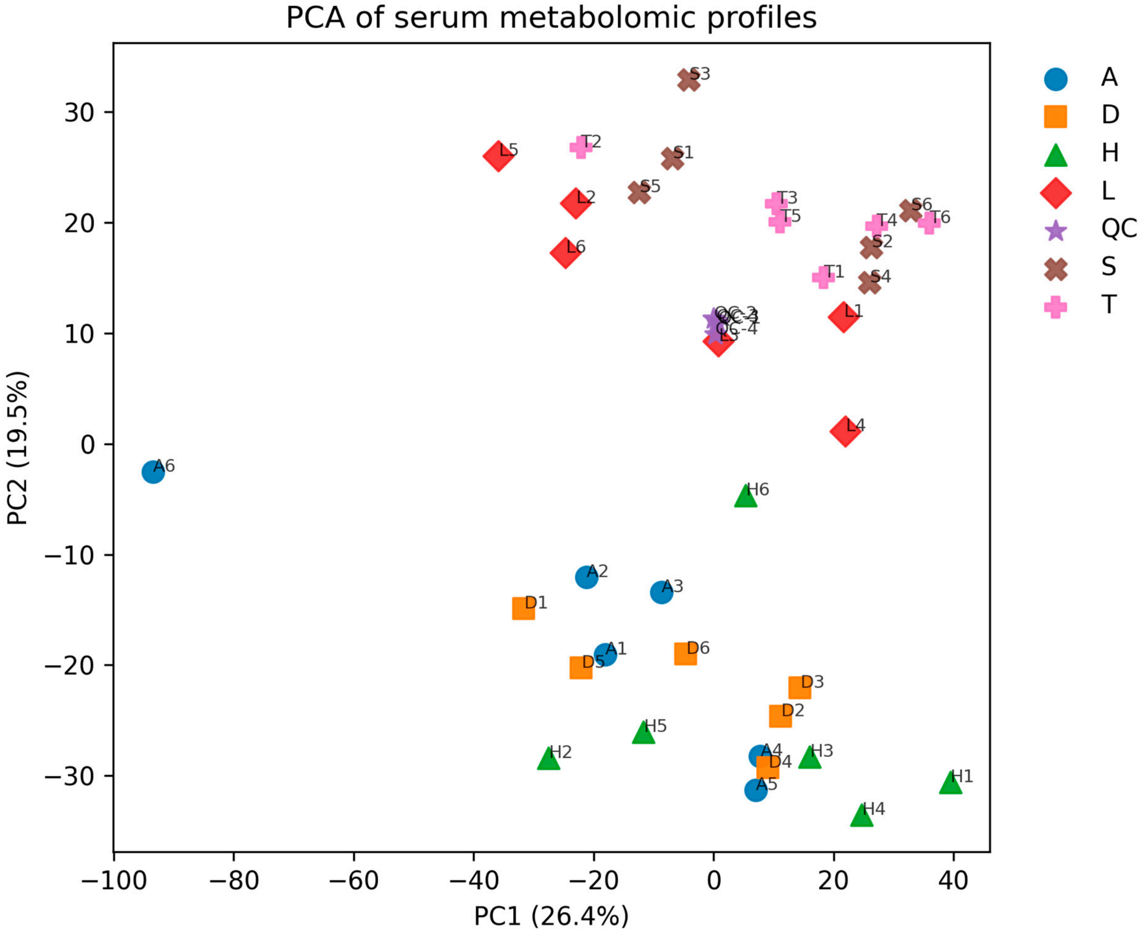

PCA including QC samples. QC clustering was used to assess analytical stability.

Supplementary Figure S2

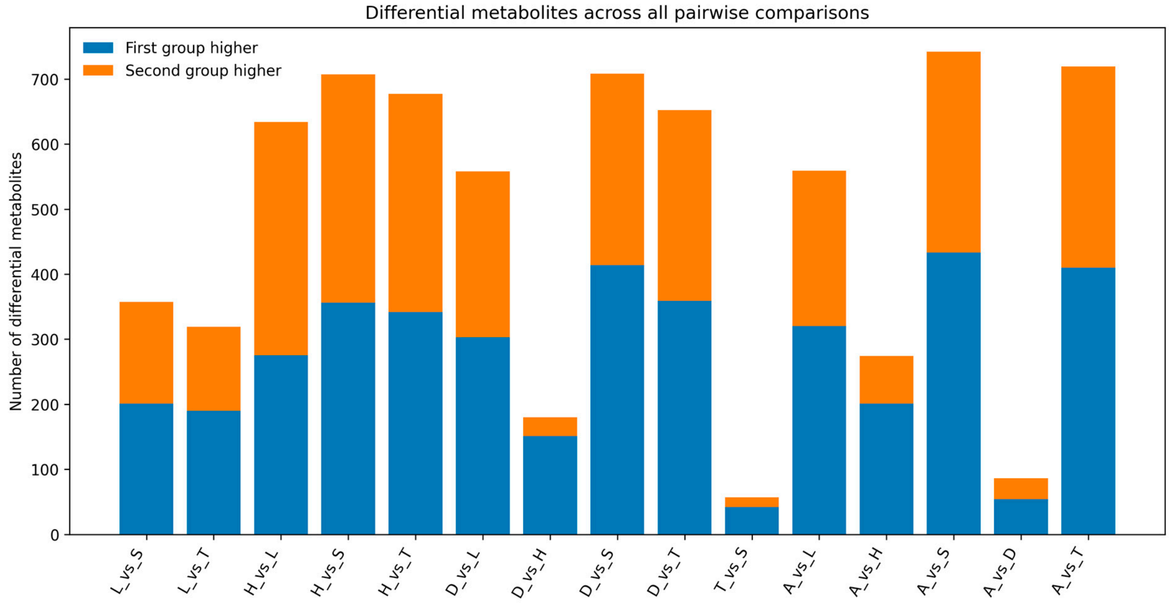

Differential-metabolite counts across all 15 pairwise comparisons.

Supplementary Figure S3

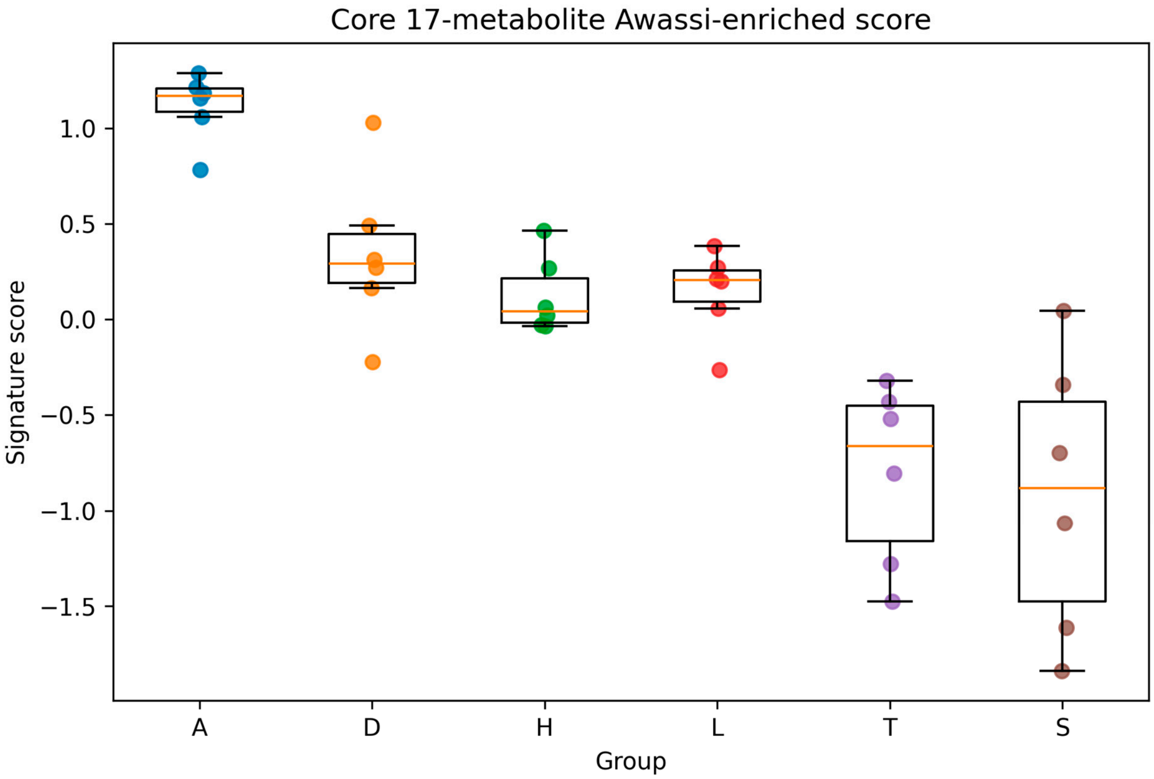

Core 17-metabolite Awassi-enriched signature score across biological samples.

Supplementary Figure S4

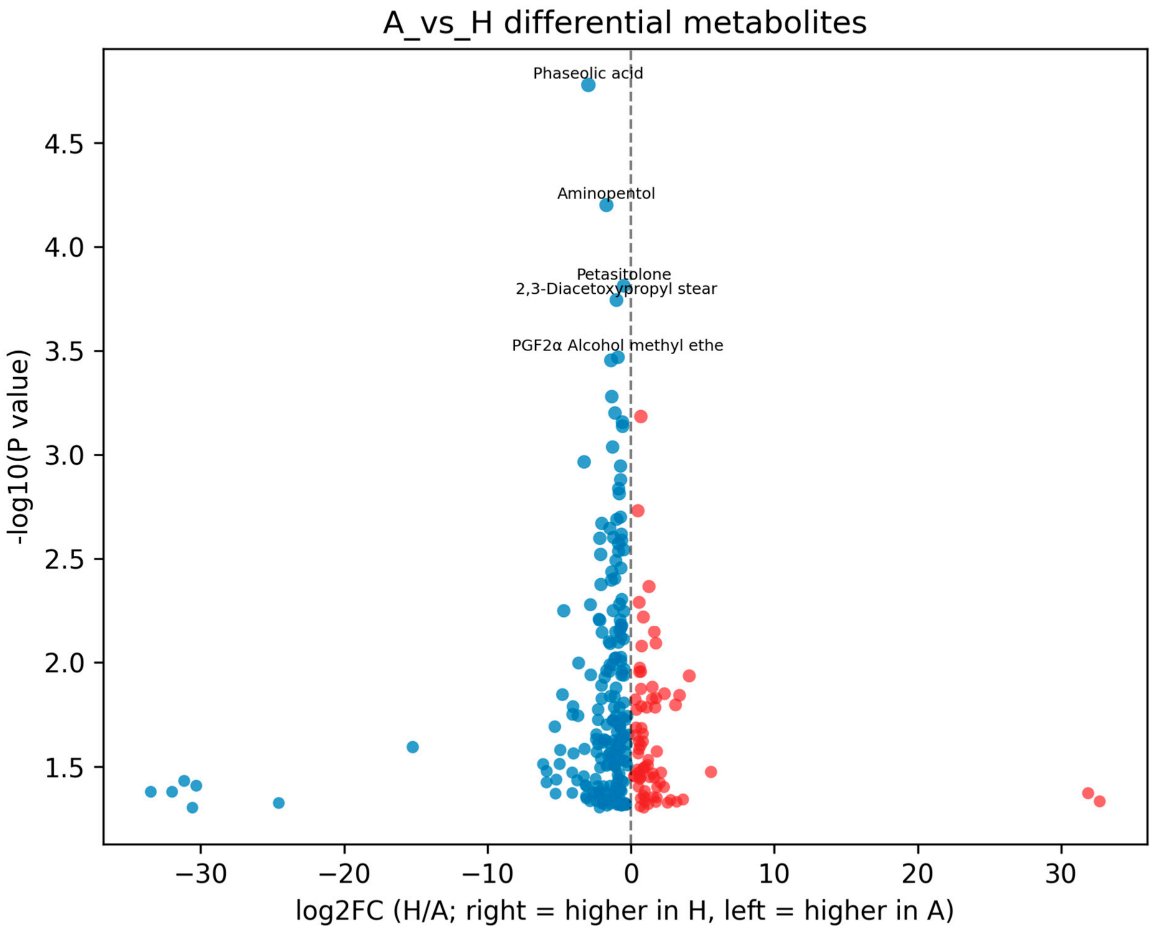

Volcano plot for A_vs_H after fold-change direction correction.

Supplementary Figure S5

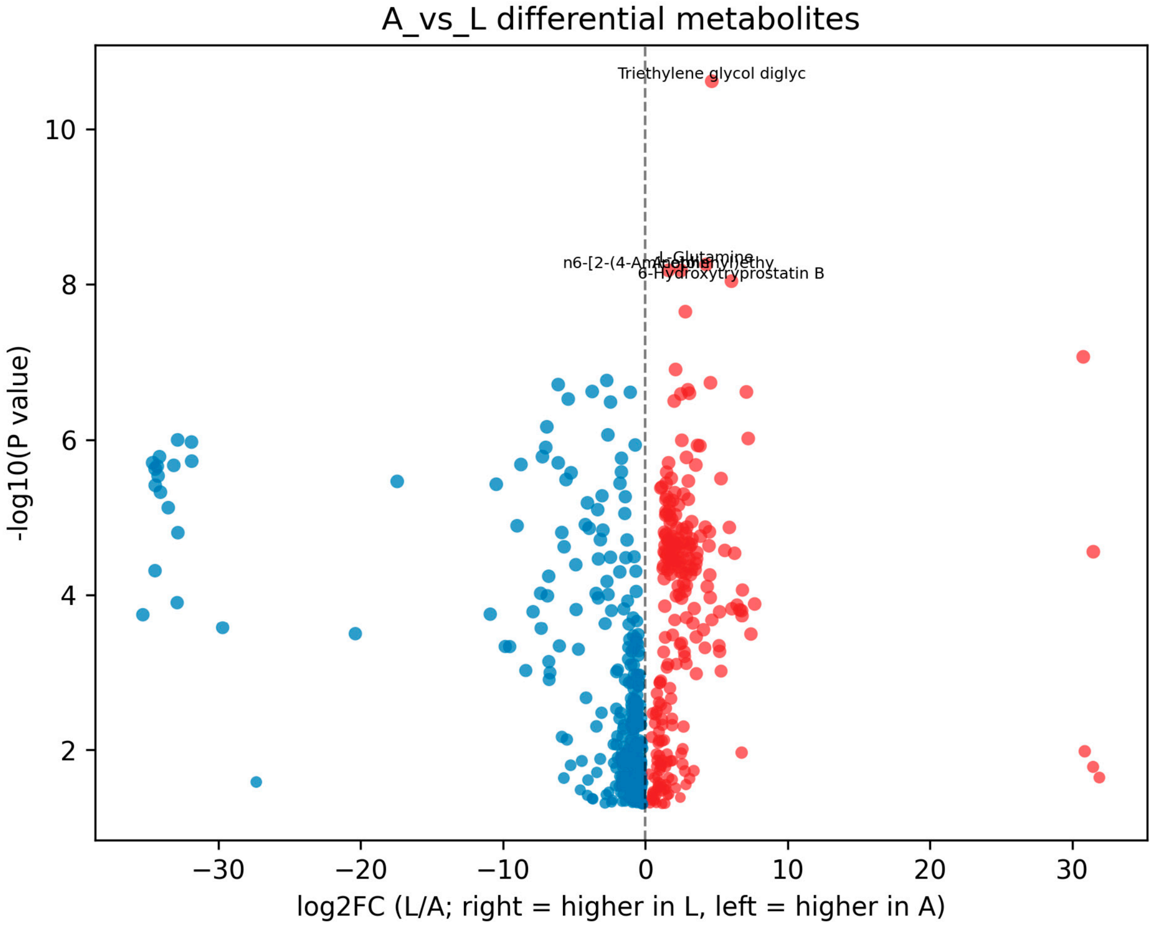

Volcano plot for A_vs_L after fold-change direction correction.

Supplementary Figure S6

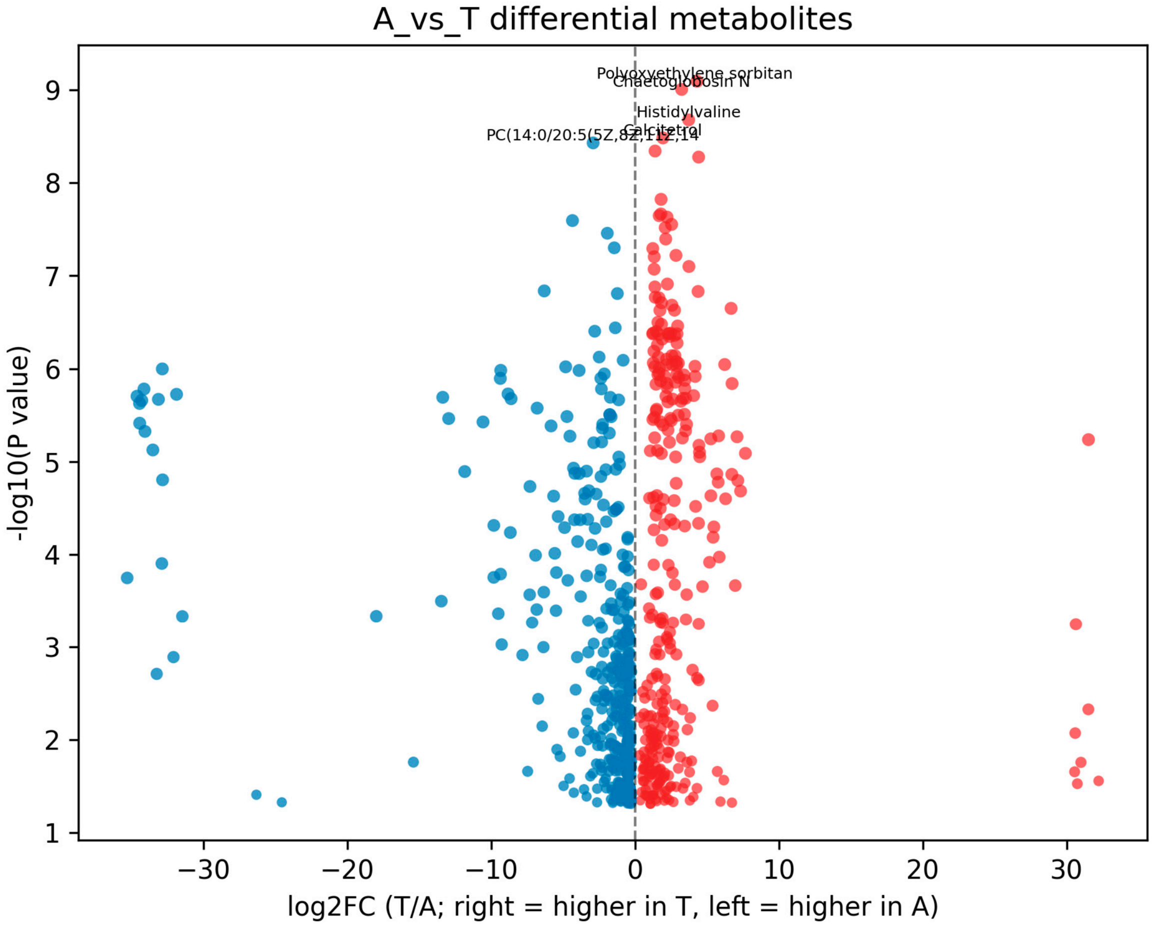

Volcano plot for A_vs_T after fold-change direction correction.

Supplementary Figure S7

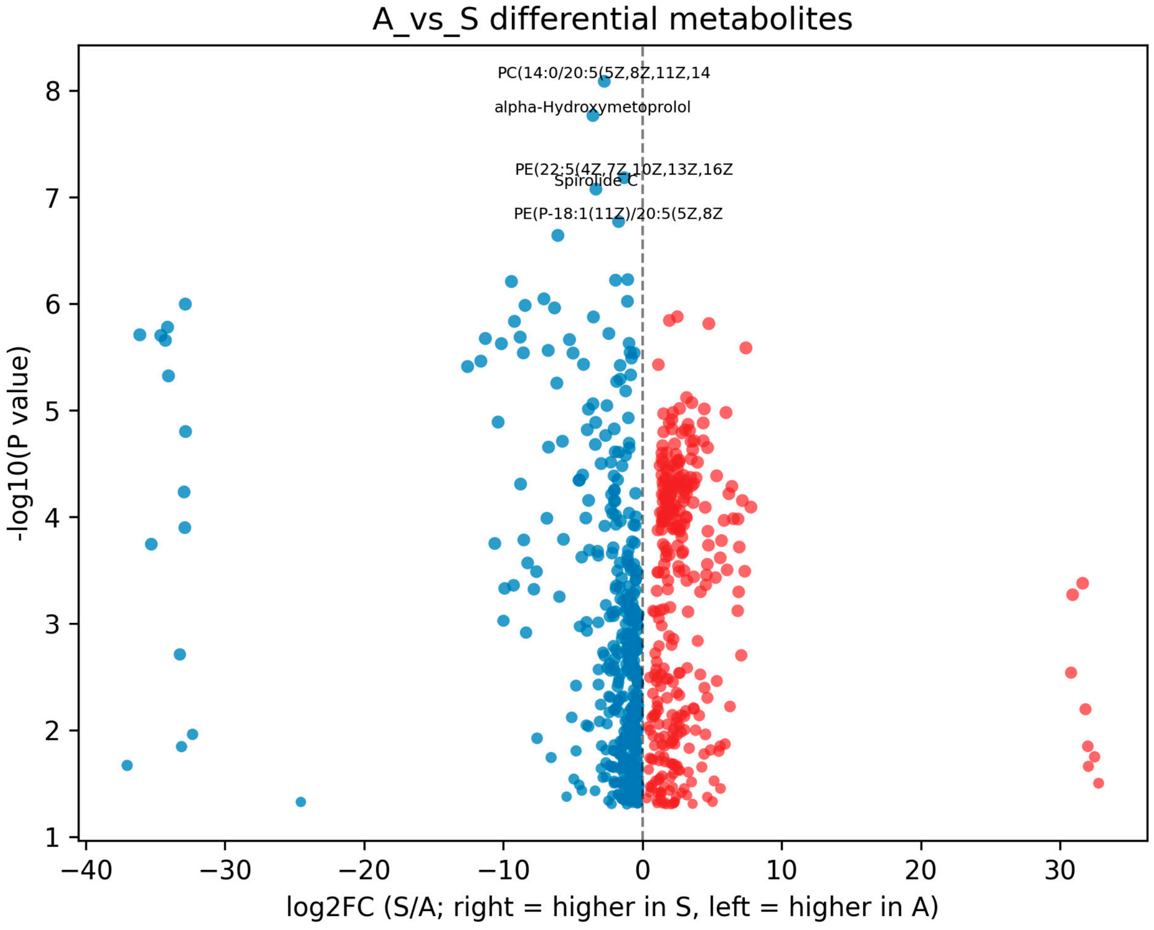

Volcano plot for A_vs_S after fold-change direction correction.

Supplementary Figure S8

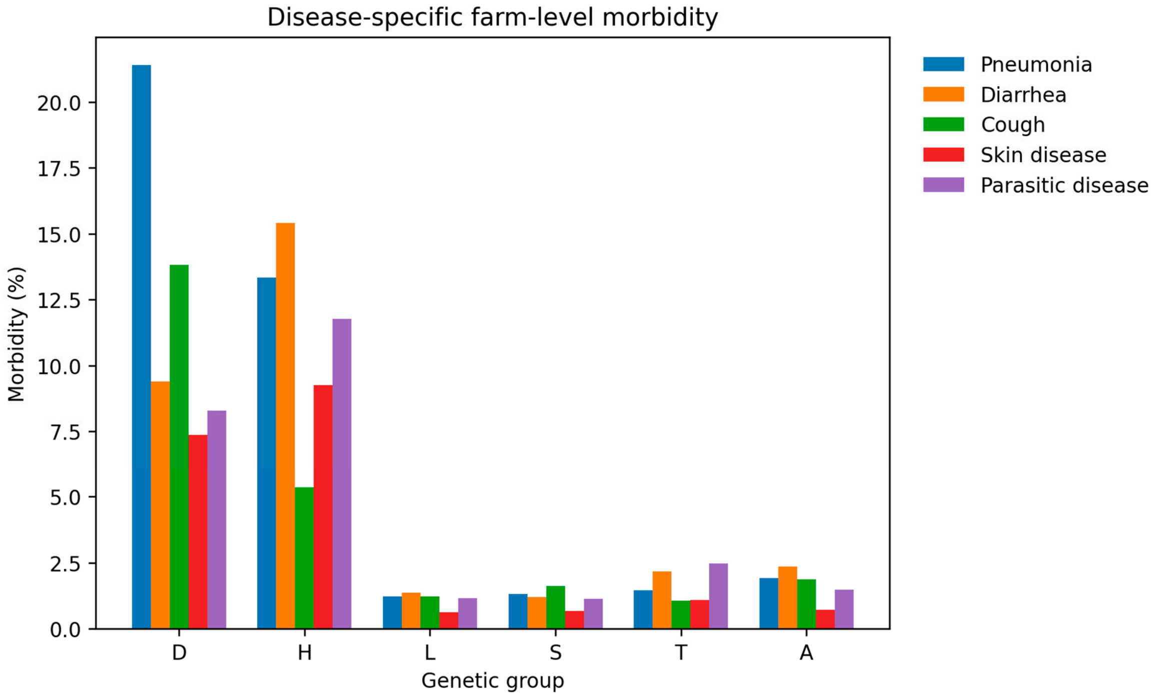

Disease-specific farm-level morbidity profiles across six genetic groups.

Supplementary Figure S9

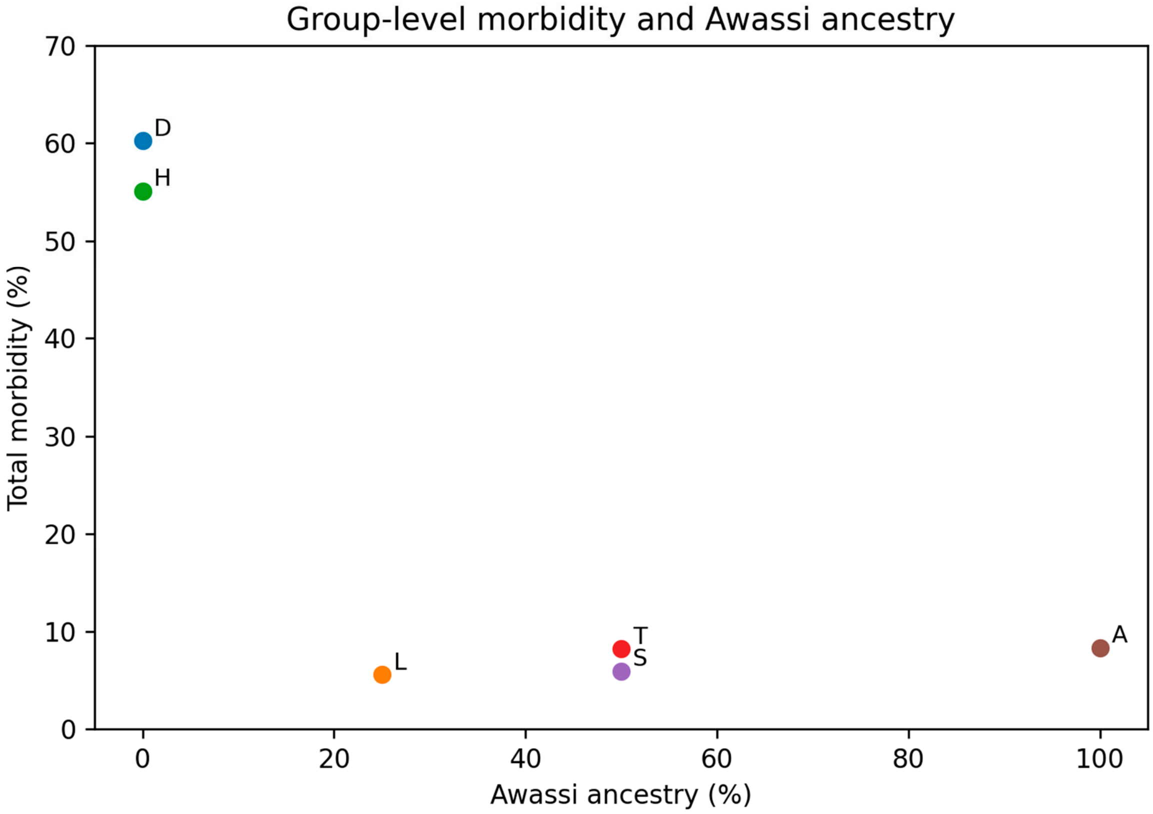

Exploratory group-level relationship between Awassi ancestry percentage and total morbidity.
