# Supplementary material for: Awassi Ancestry-Associated Serum Metabolomic Signatures Reveal Immune–Metabolic Features Related to Health Robustness in Dairy Sheep
Source: Vet Sci. 2026 Aug 11;13(8):796. doi: 10.3390/vetsci13080796 (PMC13517851; doi:10.3390/vetsci13080796)
Supplement: Supplementary file 1 [file vetsci-13-00796-s001.zip › vetsci-4415787-supplementarycaptions.pdf]

A separate Supplementary Materials document and a Supplementary Tables folder are provided with this submission package. Supplementary tables S1-S8 are supplied as spreadsheet or CSV files.

Supplementary Fig. S1. PCA including QC samples.

Supplementary Fig. S2. Differential-metabolite counts across all 15 pairwise comparisons.

Supplementary Fig. S3. Core 17-metabolite Awassi-enriched signature score.

Supplementary Figs. S4-S7. Volcano plots for A_vs_H, A_vs_L, A_vs_T and A_vs_S.

Supplementary Fig. S8. Disease-specific farm-level morbidity across genetic groups.

Supplementary Fig. S9. Group-level relationship between Awassi ancestry percentage and total morbidity.

Supplementary Table S1. Normalized metabolite abundance matrix.

Supplementary Table S2. Full differential-metabolite tables for all 15 comparisons.

Supplementary Table S3. KEGG pathway annotation table with mapped metabolites for all 15 comparisons.
